# Meetings of Societies

**Published:** 1928

**Authors:** 


### Meetings of Societies.

### Clinical Society of Bath.

A MEETING of the Bath Clinical Society was held on Friday, November 2nd, at the Royal United Hospital, Bath. Mr. A. de V. Blathwayt was in the Chair.

MR. A. S. LEVIS showed a case of **Elephantiasis of the Leg of Unknown Origin,** in which he had performed Kondoleon's operation with great success. The œdema had disappeared, and the leg was almost normal in size. The patient had returned to his work as a labourer.

DR. R. G. GORDON exhibited a cinematograph film illustrative of some abnormal gaits in children, as exemplified by some of the patients at the Bath Orthopædic Hospital. He stressed the point of the educability of many cases of cerebral diplegia which had hitherto been generally looked upon as a condition of imbecility. He cited several cases of children at the Orthopædic Hospital who, in addition to being taught to feed themselves and to walk, could also read, write, sew and typewrite.

---

A Meeting of the Bath Clinical Society was held on Friday, December 7th, at The Royal United Hospital, Bath. The Past President, Mr. A. de V. Blathwayt, was in the Chair.

DR. DUPONT (Frome) showed an **Atypical Case of Lupus Pernio** which had been seen by Dr. Henry MacCormac, whose notes on the case were read.

MR. J. S. LEVIS showed a case of a **Breast Tumour in a Young Girl.** This was thought to be either chronic mastitis or a condition of hypertrophy.

DR. VINCENT COATES showed a case which he considered to be one of **Argyria.** There had been silver medication of the eyes. This diagnosis was criticised on the score of the marked localisation of the pigmentation to the hands and the conjunctivæ, and also on account of the fact that the pigmentation of the hands rather resembled that caused by sunlight than the shade of pigmentation usually seen in cases of argyria.

MR. MUMFORD showed a case of **Marked Thickening of the Bones of the Skull in a Young Woman.** There was a history of the removal under local anæsthetic of a cyst from the upper and outer side of the right orbit, which Mr. Miles Atkinson thought had probably been a meningocele. The question of intracranial abscess was discussed, and in view of the doubtful Wassermann reaction Dr. Waterhouse suggested a provocative dose of N.A.B. and further blood examination. On account of the severe headache complained of, and the apparent smallness of the pituitary fossa in the X-ray photograph, Dr. Thomson suggested a sugar tolerance test.

He also showed a case of **Enlarged Liver in an Elderly Man** which was thought to be due to primary malignant disease.

DR. DOUGLAS MITCHELL showed the X-ray photograph of a **female patient who had had a fracture of the acetabulum with consequent encroachment of the head of the femur upon the pelvic outlet.** He gave details of the great difficulty in the delivery of this patient, who had become pregnant after the accident occurred.

DR. WATERHOUSE showed a case of **Very Intractable Stomatitis** which had resisted all forms of treatment for many months. Beyond the fact that the eosinophiles were markedly increased, there were no evidences of a constitutional cause. It was thought to be possibly of dental origin, and the red vulcanite of the patient's denture was under suspicion.

DR. DELICATI showed a case of **Amyotrophic Lateral Sclerosis with Bulbar Paralysis.** As the Wassermann reaction was positive, Dr. Gordon thought that the case might probably improve under anti-syphilitic treatment.

DR. WATERHOUSE read a short paper giving an account of five females and one male of ages ranging from 47 to 70, suffering from pernicious anæmia. Response to treatment by liver was rapid and satisfactory in all. In one patient a red-cell count of 1,150,000 and a hæmoglobin percentage of 26 rose at the end of six weeks to 3,430,000 and 50 per cent., and nine months later was 6,000,000 and 90 per cent., though a few megalocytes were still present. One patient experienced rapid and marked relief of distressing paræsthesia of the limbs. Complete chlorhydria was present in all the patients (four) to whom test meals were given, and in another was known to have been present thirty years previously. A history of sore tongue was obtained in four patients, and in one of these had been severe enough to prevent the taking of solid food. Three of the patients complained of loss of flesh. In only one was the spleen enlarged.

